# Engagement in rapid public health research among young people from underserved communities: maximising opportunities and overcoming barriers

**DOI:** 10.1186/s12889-024-19762-6

**Published:** 2024-08-14

**Authors:** Sarah Denford, Lydia Holt, Rosie Essery, Joanna Kesten, Christie Cabral, Dale Weston, Jeremy Horwood, Matthew Hickman, Richard Amlôt, Lucy Yardley

**Affiliations:** 1 Population Health Sciences, Bristol Medical School, University of Bristol, Priory Road Complex, Bristol, BS8 1TU UK; 2 School of Psychological Science, University of Bristol, Bristol, UK; 3 The National Institute for Health and Care Research Health Protection Research Unit (HPRU) in Behavioural Science and Evaluation, University of Bristol in collaboration with UK Health Security Agency, Bristol, UK; 4 Academic Unit of Psychology, University of Southampton, Southampton, UK; 5 The National Institute for Health and Care Research Applied Research Collaboration West (NIHR ARC West), University Hospitals Bristol and Weston NHS Foundation Trust, Bristol, UK; 6 Behavioural Science and Insights Unit, Health Security Agency, Porton Down, Salisbury, UK

**Keywords:** Diversity, Equity and inclusion (DEI), Young people, Co-production, Research practice, Public health

## Abstract

**Background:**

Inclusion in public health research of young people from low-income households and those from minority ethnic groups remains low. It is recognised that there is a need to change the way in which research is conducted so that it becomes more inclusive. The aim of this work was to identify novel and innovative ways to maximise recruitment and inclusion of diverse participants when doing co-production within very short time frames for emergency responses.

**Method:**

We conducted interviews with young people from low-income and minority ethnic backgrounds, and members or leaders of groups or organisations supporting or representing young people from underserved communities.

**Results:**

A total of 42 participants took part in an interview. This included 30 young people from low income or minority ethnic backgrounds and 12 community leaders/service providers. Of the 30 young people, 26 participants identified as female and 12 participants identified as being from a minority ethnic background. Participants discussed a number of interrelated barriers to research involvement and identified ways in which barriers may be reduced. Prejudice and discrimination experienced by young people from underserved communities has led to substantial mistrust of educational and governmental establishments. Rigid and unfamiliar research practices further limit the involvement of young people. Four themes were identified as ways of supporting involvement, including: making opportunities available for young people, adaptations to research governance, understanding and acknowledging challenges faced by young people, and ensuring reciprocal benefits.

**Conclusion:**

This research explored barriers to engagement in rapid public health co-production. Working with communities to co-produce rapid recruitment and research procedures to suit the needs and the context in which young people live is necessary.

**Supplementary Information:**

The online version contains supplementary material available at 10.1186/s12889-024-19762-6.

## Introduction

To develop acceptable, relevant and accessible public health interventions and messages that address the needs of the target audience it is crucial that those who will be receiving the interventions are involved in their co-production [1–3]. However, the nature of public health and public health emergencies means that interventions frequently must be developed and deployed very quickly. While there are established methods for inclusive co-production [1, 4], these often depend on much longer timescales than are available when responding to public health emergencies.

When developing interventions, specific populations may be involved in multiple ways [5]. Often, people are invited to share their views and experiences as a participant in qualitative interviews. Other individuals may be invited to join the research team as someone with lived experience of a particular condition or context. In this case, the individual may be considered a patient or public involvement (PPI) representative and will be involved in activities that inform the research and the way in which the research is conducted. Others may be invited to be involved in the co-production of the intervention materials. Whilst the term co-production is defined in different ways to refer to a range of activities and ways of working with the target audience [6], it tends to involve researchers and members of the public working together to achieve a shared outcome (e.g., the development of intervention materials). Different approaches to recruitment and engagement may be more appropriate depending on whether the aim is to recruit individuals as participants or include them in PPI, but many common barriers to engagement prohibit public involvement altogether [7].

Despite guidance from the National Institute for Health Research stating that everyone should be given the same opportunity to take part in research (as PPI and participants), regardless of race, age, gender, or ethnicity [8], inclusion in research of individuals from low-income backgrounds and those from minority ethnic groups remains low [1, 7, 9]. Inclusion of young people from these groups is even lower [10]. Individuals from these groups are considered to be “underserved” and by definition not represented in research, and are less likely to respond to, or engage with, healthcare interventions than the general population [11]. Whilst this has always been the case, the COVID-19 pandemic exposed an urgent need for academics to identify ways of doing better, more inclusive research [12] so that it is accessible to those who are currently excluded.

Our research group recently published a research paper in which we propose a novel framework intended to provide a focus for investigating new ways of rapidly developing effective interventions by combining co-production methods with large scale testing and real-world evaluation [13]. A recent scoping review identified a lack of such approaches in the literature [14]. The first step in this process is to co-produce inclusive and reciprocal ways of working with underserved groups when interventions are rapidly required.

### Aim

The aim of this work was to identify novel and innovative ways to maximise recruitment and inclusion of individuals from underserved communities as PPI representatives or participants when developing interventions within very short time frames for emergency responses.

In particular, we wanted to:

1. Identify barriers to participation and involvement in research among underserved young people.
2. Identify possible methods of recruitment and engagement with underserved young people for rapid intervention development.
3. Explore perceptions of and barriers to the use of strategies for recruitment and engagement for rapid intervention development.

Design and methodsWe conducted qualitative research using semi-structured interviews with young people from underserved communities, and staff or volunteers working in groups/ organisations supporting or representing them (see interview schedule supplement).

## Method

### Inclusion criteria

#### Setting

This research was set in Bristol in the South West of England, the largest city in the South West of England. In 2021, 18.9% of the population identified as being from a minority ethnic community, and 15.6% of the population were between the ages of 16 and 24 years [15]. Bristol has some of the most deprived areas in the country, with approximately 15% of the population living in the most deprived of areas in England [16].

### Participants

#### Young people

Participants were eligible for inclusion if they were between the ages of 16 and 25 years and from one of two groups who are often underserved in health and research (1) minority ethnic groups, and (2) people who are socio-economically disadvantaged/ unemployed/ on low income.

We defined socio-economic disadvantage as those who were unemployed, receiving universal credit, or receiving minimum wage, and living in areas that are in the most deprived deciles in the South West of England according to the Index of Multiple Deprivation (IMD) 2019.

#### Representatives of young people

People working with young people from underserved communities were eligible to take part if they worked or volunteered for an organisation that aimed to support or represent young people from underserved communities in the South West of England.

### Recruitment

#### Young people

There were two initial routes to recruitment of young people: (1) Study advertisements were shared on social media (Facebook, Instagram, WhatsApp) and used hashtags, groups, and targeted advertising to facilitate recruitment of the target population. (2) We invited community group leaders to share study advertisements with members of their community on our behalf (e.g., via notice boards in hostels, newsletters etc.).

We used an iterative approach to data collection and analysis so that the insights from early interviews were used to inform the development and adaptation of subsequent approaches to recruitment and engagement. Based on feedback from early interviews we then used a snowball approach to identify other relevant participants who were willing to speak to us, but who would not otherwise take part in research (e.g., through previous participants sharing adverts with their peers via social media channels).

#### Representatives of young people

Representatives of young people were recruited through existing contacts at Bristol City Council who were able to connect the research team with groups supporting the most vulnerable young people in Bristol and surrounding areas.

### Procedure

Participants had the option to register their interest in multiple ways. For those recruited through study advertisements shared via social media, adverts directed interested participants to an online sign-up page (hosted by Qualtrics) where they were invited to read a participant information sheet and complete a pre-screening questionnaire including demographic and contact details. The researcher’s telephone number was provided for people to call, text or WhatsApp for those who could not, or chose not to use the online sign-up page. To mitigate against issues of digital exclusion, whenever appropriate, potential participants had the option to ask community service providers to contact the research team on their behalf if they preferred.

Purposive sampling was then used to recruit participants, aiming for diversity in gender, age, ethnic and socioeconomic backgrounds. Selected participants were contacted by the research team via email, phone or text (as per participant preference) to arrange a time for an interview to take place. At this stage, all participants were asked to complete an online consent form (hosted by Qualtrics) consenting to take part in the research. Telephone or paper consent was obtained by those preferring not to use the online form. Those not invited to interview were contacted by text or email and informed that they had not been selected to take part.

Initially, interviews were conducted by SD via Teams or phone. Based on insight from these interviews, subsequent participants had the option to take part in the study in person, via Text, WhatsApp or Facebook Messenger. An amendment to the ethical approval was submitted and approved by the ethics committee to permit this change.

A topic guide with open ended questions was developed for this study (supplement 1) and explored how young people from underserved communities viewed approaches to recruitment and engagement, with a particular focus on the perceived relevance and appropriateness of these approaches. We specifically focused on potential problems or barriers and aimed to elicit suggestions regarding how we may improve recruitment and engagement in the future.

Participants who work with young people were asked to consider challenges associated with engaging with young people from underserved communities, how they currently communicate and engage with the young people they work with, who these communications are likely to reach (or miss), and recommendations for improving the way in which research teams work with young people.

All participants received a £25 shopping voucher of their choice as reimbursement for their time. Ethical approval was granted by the School of Psychological Science Research Ethics Committee at the University of Bristol (ethics approval code 10595).

### Data analysis

Data were analysed using reflexive thematic analysis [17]. All data, including transcripts of recorded interviews, notes from a group interview (not recorded as per the wishes of the group), and written messages (including WhatsApp, Facebook messages), were anonymised and added to the software platform NVivo. Data were then coded through line-by-line coding by the lead author. A preliminary coding framework was developed by the lead researcher on the basis of this initial coding and discussed with the research team. The remaining data were coded in accordance with the framework and checked by a second author. Adaptions to the framework were made as necessary. Once all data had been coded, the team noted that the barriers include opportunity barriers, motivational barriers, and perceived capability barriers, and the COM-B model [18] was identified as a useful way to organise the themes. The analysis continued during the writing stage [19] as the team considered and presented issues and solutions as per the aim of the project.

## Results

A total of 42 participants took part in the research. This included 30 young people (between the ages of 16 and 25 years) from underserved communities and 12 people who work with young people from underserved communities. Of the 30 young people, 26 participants identified as female and 12 participants identified as being from an minority ethnic background.

A total of 12 participants were recruited through hostels / community groups, and 18 participants were recruited through word of mouth/snowball sampling (including participants who responded to adverts shared by peers on social media).

Participants shared their views through: online or telephone interviews (eight participants), in person (four participants), group interview (four participants), WhatsApp group chat (six participants), Facebook Messenger group chat (four participants), individual synchronous WhatsApp chat (two participants) and individual asynchronous WhatsApp chat (two participants).

All 12 of the participants who work with young people took part in an online or telephone interview.

### Results of the thematic analysis

Participants discussed a number of interrelated barriers to involvement in research and identified ways in which barriers may be reduced. Below we present four themes that describe recommendations for facilitating engagement with young people:

- Making opportunities available;
- Adaptations to research practice and governance;
- Understanding and acknowledging context faced by young people;
- Ensuring reciprocal benefits.

Illustrative quotes are presented below from young people (YP) and people from organisations who work with young people (PWYP). Within quotes, use of […] indicates that text has been removed because it was not relevant. Brackets ( ) are used to show that text has been added for clarity. This approach was frequently applied to data obtained through text messages and group chats in which people were simultaneously sending replies in response to questions, and thoughts and ideas often spanned multiple messages.

### Making opportunities available and accessible

A key barrier to involvement among young people was that opportunities were not made available to them. This includes both short term opportunities to be involved and share their opinions, but also longer term and sustained opportunities where people can develop their skills. Many young people spoke of research adverts not being sufficiently relevant, targeted, inclusive, or shared in locations frequented by themselves or their peers. Indeed, one group of young carers, who did not wish to be recorded, stated that one of the key reasons for not noticing or responding to adverts was that adverts are very often subtly suggesting that they are not eligible through where and how they are shared. Adverts were perceived as portraying the message that the opportunity is not intended for them through the location, model, text, style, images, or phrasing. The group reported that attempts to include images of someone who “looks a bit like me” on the advert, without any other attempts at inclusivity, were not sufficient. Inappropriate or complex language often made opportunities inaccessible for people who are not familiar with public health or research practices:

I don’t take part [in public health research] because I don’t even ***ing know what it is. (YP).

Most disadvantaged young people often lack a space in which they feel they belong. This makes it difficult to identify appropriate locations within which to advertise opportunities:

See [advertising research opportunities to very deprived young people is] tricky because, especially thinking about the young people that I work with, where do they hang out? Outside of a shop? Or just, they don’t really hang out because you need like money to hang out and they’re in the outskirts, just hidden.…(PWYP).

Whilst community groups are often approached to aid recruitment, it was noted that relying on this approach alone will lead to a subset of the population being excluded, and that those who do attend community groups may be atypical of the wider population:

I don’t belong to a community group or wouldn’t know where the nearest one is. So you’re again, you’re just kind of getting that particular group who actually are probably not that difficult to recruit… they’re always a certain sort of person who would probably have very different views to the sort of people who wouldn’t go to those groups (YP).

In order to improve recruitment, young people highlighted the need to create advertisements that are appropriate and inclusive for young people. This needs to take into consideration where young people are, how they speak, and what inspires/motivates them. Including the voices of young people in the creation of materials is essential:

Yeah, when everything is like, and you read it and you’re like, oh no, you know, like someone over 40 has turned around and gone through like a list of BuzzFeed’s top ten slang of 2022 (YP).

In response to feedback, study advertisements were modified to include images of the areas in which we were aiming to recruit. When prompted, young people reported that inclusion of local images made them feel more confident in the legitimacy of the research.

As young people often reported a lack of “space” and connection to their community, it was felt that the best approach to rapidly recruiting young people is through trusted word of mouth. In response to this, at the end of interviews, we asked young people if they could share a study advert with a peer who may have a different perspective to themselves. Following this, a total of 18 young people were recruited through word of mouth. This approach relies on identifying potential participants who may be willing to engage with research teams and who can provide critical access to a wider, diverse range of individuals beyond those involved with community groups (particularly if incentivised).

As soon as you get one young person or a couple of young people who have spoken to you and your colleagues, then they’ll say to their friends, oh I’ve done this, have you done that and you know that if I spoke to the guys now and said, oh I spoke to ((name)), they would be more likely to talk to you (YP).

Add in a refer a friend bonus that you know these, so you know do your interview for 25 quid. If you get a friend to do it, they get 25 quid and you get another 25 quid (YP).

Importantly, those recruited via word of mouth were more likely to report having been put off by complex research procedures, less willing to take part in a formal interview, and more willing to share their views via the approaches outlined below.

And like I said, ((name)) did message me and say, oh can you do this [WhatsApp interview] and yes, why not? Absolutely fine. But if she said, can you go and do a group thing or like talk on the phone? I probably wouldn’t. I would have said oh no, I’m busy (YP).

Identifying the individuals who will engage is not always straightforward, and increasing the diversity within research teams and other organisations is essential:

More diversity within our workforce, and that’s the only way to do [better engagement with diverse communities], to start employing people that are from those communities (PWYP).

### Adaptations to research practice and governance

Standard research practices, such as consent procedures and recording of interviews, were often unfamiliar to the young people. Adaptations to research governance, with the aim of making participation less intimidating could be important. Furthermore, there was a clear sense from participants that a divide exists between themselves and research or public health teams.

It’s such a barrier isn’t it [the University] just being a big organisation, like a government organisation. Straight away like oh, that’s not for me. (YP).

Many young people described themselves as lacking skills, training and intelligence to engage in University-led research. Young people were worried about their ability to usefully contribute because they did not understand academic terminology and complex research processes:

Because is it when people are a bit unknown, maybe people think they’re not clever enough or it’s… it sounds awful, but they look at University of Bristol and do they think, actually am I a bit stupid for that? I think maybe a big thing is people don’t think they’re clever enough to do it. Maybe they’re worried about what they’re gonna have to do (YP).

Lengthy and complicated consent procedures and information sheets exacerbated anxieties and further reduced willingness to take part.

‘I know I’ve sort of applied to do different things and then it’s come and I’ve thought oh God, no it’s way too complicated (YP).

Like the kind of people you’re looking for… kind of make sure your research is… kind of easy for people to do. There are a couple of times, kind of like after every second line, trust me, I got half-way… point… I was lost… (YP).

In order to facilitate engagement with young people, research teams will need to be willing to keep processes simple.

Keep the question simple and straightforward… smart, intelligent, not everybody is like you, so there are some people like that, that will find a very simple question, they… definitely, you have to keep the question very simple (YP).

It was often felt that the best way to obtain the views of young people is to simply ask them what they think – without off putting long and complicated information sheets and worrying research procedures, which often prevented young people from getting involved in research. Ethics committee regulations did not permit us to change our consent form during the study. However, we were able to ask for feedback on the consent statements, with nearly all young people reporting that they would feel more comfortable signing simpler consent forms. Indeed, most successful attempts to engage with diverse groups appeared not to involve ethical procedures at all:

I think I’d be comfortable with the shorter [consent statement]… [standard consent procedures may be important, but] only if they understand them (YP).

I didn’t have to go through their ethical approval. So I was able to do things like spend time where I knew I wasn’t hearing from… So it was going to where they were…. I was able to do that and they didn’t want me using a Dictaphone because they said it made me seem like a police officer. And the research that wasn’t discounted because they hadn’t signed consent forms or I hadn’t got the Dictaphone on”(PWYP).

Providing questions or topic guides to participants in advance of the interview was reported to make people feel more comfortable taking part, and provided people with the opportunity to think about their answers in advance:

People who don’t work in a University may not have any idea what to expect… So yes, I think being aware of [what questions will be asked] and also, it’s not like a scary thing is it? But at least you’ve got some idea of what you’re in for… you’re not so apprehensive about, oh God, they’re gonna ask me a load of stuff, I’m gonna just go um, um sorry, I don’t know. (YP).

Like with anything I’d then probably have a think about it and then you’d probably get better, I know from me, that would get better feedback, like actual like, oh, I’ve had a think about this, I’ve got actual opinions, I’ve had a chance to kind of. (YP).

If possible and appropriate, additional support from trusted individuals could also reduce anxiety among young people:

I do get some young people where it’s the first time they’re going to be talking to a group of people about a particular thing and it is really, really daunting. So I’ll usually do a little session on Zoom with them first and then they’ll come to a rehearsal where they can meet all the other young people that are getting involved, and I’ll be in a breakout room with them to help facilitate the conversation and stuff, so it’s a lot of hand-holding sometimes. Yeah, it can be really daunting (PWYP).

Making an effort to make the activities fun and enjoyable is also likely to help sustain involvement:

Some of my mates wouldn’t take part in something like this but are always taking part in stupid online quizzes and stuff. You see it all the time. They’d never agree to do a survey or anything but they’d give away their bank details on a quiz to find out what flavour crisps they are… I bet you could get tons of info on people if you get a bit creative. You know, ‘Do you like this or this? Or do you prefer this?’ And then you tell them what sort of covid sceptic they are (YP).

### Understanding and acknowledging the context and challenges faced by individuals

Many disadvantaged young people were faced with considerable struggles on a day-to-day basis. Overcoming these challenges took priority over participation in research:

A lot of them are struggling to live day to day. Like they’re just trying to stay alive, like you know they’re trying to get food, they’re trying to like get by. So for some of them, they might just feel like they haven’t got the energy or the time to engage in something that they don’t fully understand (PWYP).

Mental health issues have a huge impact on the lives of young people and their confidence, and this had been exacerbated through COVID-19 lockdowns:

I was reading the other day that 1 in something crazy stupid like 1 in 2 or 3 of us have mental health problems like anxiety or whatever at some point. So maybe there are lots of people that would just find [taking part in research] too stressful and their anxiety would stop them… because I work with people who have mental health challenges I can really see that (YP).

Many people had previously been exposed to discrimination and had been let down by the system and by professionals at many levels. Trust in the government and related organisations were low:

I think people hate the government. They do you know. I think they don’t trust a word that comes out those people’s mouths and I think particularly for groups of people who’ve been left behind by this government, whether they’re white or black, you know in [area] right now, it’s where I grew up. And it doesn’t matter whether you’re white or black here, you’re poor. That’s the signifier that defines where your life is gonna go (YP).

There was similar evidence of mistrust in science, scientists and Universities:

Yes, because science has never had political agendas lmfao (laughing my f***ing arse off’) (YP).

The problem is the arrogance. Scientists speak in absolutes and make fun of people who disagree with them (YP).

No, it’s because the scientists in question are usually on a company payroll. Kinda like tobacco company scientists claiming smoking doesn’t cause cancer (YP).

Many people did not feel connected to their communities or to society, thus did not have a strong sense of social responsibility:

I don’t think that [young people are] always rejecting society or their community. I think they just don’t have a link to it, so [helping with research] isn’t worth it for them… (PWYP).

Systemic issues within society create a situation in which people feel excluded at multiple levels:

When you think about how oppressive our society is for them, actually, the odds of them being able to find the headspace, the time, the resilience to engage in public health research, it’s wildly… I mean, you know…(PWYP).

Following the introduction of policies during the COVID-19 pandemic, some young people reported not wanting to engage in any research that could support a political agenda they may not agree with:

We don’t want to be like maybe speaking to people and later on it backfires on us (YP).

Research teams may need to make efforts to demonstrate neutrality when recruiting, for example, through selective use of logos on research adverts:

I find that quite sort of obvious and you feed [research findings] to the government and that’s the Conservatives at the moment but you would do the same to whoever else. But I can see how potentially maybe for other people that might not be immediately obvious. So yeah, definitely sort of using independent and neutral [approaches].

Working with trusted individuals, for example, those who work with the community, to support the development of relationships with community members may support involvement:

For sure, yeah, yeah, cause we’ll be seeing them in our groups and for one-to-ones anyway, so we’ve already got that time with them, so we can just use that to be like, why don’t you come along to this thing, here’s some incentives, come on, we’ll book you a taxi, and get them in there. Whereas I think, as an external organisation, reaching out to groups of young people, I can imagine it’s difficult. (PWYP).

Small adaptions to research practices can make research opportunities more welcoming for young people:

I know from feedback, I’ve been told that they prefer to meet me just out for a coffee ‘cause that’s what people do, rather than meeting in the office, where they just feel like, oh I’m a service user (PWYP).

For research about sensitive topics, sensitivity is needed to support people to trust the researcher and to open up:

Get a meal deal and go for a walk or something like that or meet in a cafe or maybe something like. What’s really useful is getting the bus or driving together somewhere because then it isn’t so like a kind of interview where it feels quite intense because then they just won’t talk (PWYP).

In many cases, people did not have access to the internet or sufficient credit on their phones to take part in remote discussions. Providing people with credit or access to phones would be essential:

They let you use the phone here at [service] sometimes, but yeah if I had no credit (YP).

For those with limited access to the internet, vouchers for coffee shops provide young people with the opportunity to partake in every day activities and benefit from free Wifi that is often available:

I have given vouchers to a young person… There was a young person I was working with who needed to log on to do their e-learning and they didn’t want to come into my office to do it ‘cause they would have to do it while I was there and yes, just timings wouldn’t work out very well. So I was giving them vouchers to go to the cafe, you know one that you can buy vouchers for like a Starbucks or a Costa or something and then they can use the free internet there. And so then they just again, just felt like they were a student, that normal students would do and they would just go buy a drink, stay there for four hours on one drink and yes, just do it like that. (PWYP).

In other cases, people lacked a safe space within which to speak – particularly around topics that may be sensitive:

With an LGBTQ group, they might have the tablets, the computers, the technology, but they might not have the safe space in order to talk about that, if they’re not out to their family or if they live in a very transphobic household – they’re not gonna be able to speak honestly (PWYP).

Giving people the option to take part in research in a way that suits them will substantially reduce access barriers. This could be face to face, remote, or written, but must be tailored to suit the circumstances of the individual:

I think also like we live in like a world where people don’t talk now. You know if your research is primarily on the phone, like some of the other guys who I work with who are like 20/21 years of age, they don’t ring people like. They just message or things like that. No one phones anyone anymore and stuff like that (YP).

Right now I’m sitting on the floor with the baby on my lap. It’s not ideal, but I would never have been able to speak to you if it had to be at a particular location or at a particular time (YP).

In addition to face to face or phone, offering people the option to take part via WhatsApp or messenger reflects the communication styles of young people, and can also reduce anxiety associated with speaking on the phone or in person:

I always sometimes can get anxious with phone calls and things like this sometimes, so actually having that kind of like text format and things helps to remove that because you’ve got that little bit of a buffer of like time to be like, oh, I’ve got to read that or take a moment to process it and go back on your own terms rather than, and also I just like the convenience of text(YP).

For people who do not keep traditional hours, using asynchronous approaches will give people the flexibility to respond at a time that suits them:

I’ll often get emails at maybe like two in the morning… it might be difficult for the flow of conversation for you to be able to have that, but maybe with that flexibility of like we could keep replying and just see what you get from it rather than it needing to be within a time slot. It’s like let’s have the – like the flexibility of letting the conversation carry on over days rather… Just because that’s what tends to happen with the young people. I’ll be texting them and then I won’t get something for two days and then (PWYP).

### Reciprocal benefits

A primary concern among young people was that taking part in research would have no benefit for them or their peers. Many thought that their contributions would fail to have impact and that no one would be listening.

I guess the older you get, the less you’re probably keen to engage ‘cause you sort of feel that you’re not, by the time you’re in your 20s, that you’re not listened to by I guess, government it’s seen as. (YP).

I suppose the obvious [barriers] are that their contribution might not make a difference and the request for help is lip service (YP).

Guidance recommends that research should address the needs and priorities of marginalised communities, and this may increase engagement:

And probably like who’s it gonna benefit. Do you know what I mean? Like why am I doing this? Not just for like a voucher, but like what services and things like that is it gonna benefit? Like who in the future and people like that? You know who is it gonna help really? (YP).

However, during public health emergencies, national policies may differ from local community priorities. This results in a situation in which research teams are attempting to recruit people to co-produce interventions to address national (or international) public health priorities, while individuals and communities continue to face a range of other very serious challenges that are more important to them. Motivation to take part in research in these circumstances is likely to be low, and attempts to force topics can be damaging to any established relationships already developed:

Well, yes, it was a funny thing because our organisation wanted us as frontline staff to be talking with the young people about their injections and their [COVID-19] vaccinations, and their like social responsibility, and sometimes it was quite conflicting as somebody that has the relationship with that young person because, as you’re saying, it might seem quite far from what they actually care about. And of course, there’s the whole world is caring about it, but for this person, it’s like one tiny thing amongst like everything that’s going on. They’re probably not caring about whether they’re vaccinated, if they’ve got like a gang after them, which sounds silly, but you know it was like the reality (PWYP).

Ensuring participants are reimbursed for the time they spend contributing to research is essential. Importantly, payment must always be offered in a format suitable for the population in question:

So making sure that young people are paid for their time, and that can mean different things. That can mean money, if you’re working with asylum seekers, paying asylum seekers can have a real detrimental effect on the process of asylum, so it’s about food vouchers, it’s about finding out what incentive is best, but making sure that that is fair (PWYP).

During the pandemic, one of the groups I spoke with provided Dominos to people – this was really appreciated by the young people who were seeing everyone else have deliveries, but were unable to afford it themselves. Some of these clearly still require funds – but perhaps not so obvious as cash or vouchers… pizza works really well for young people. Just all of these things, you know warm food in a belly does so much for hungry young people (PWYP).

However, financial reimbursement may not always be sufficient to support engagement:

You know I think because their lives are quite chaotic that they might say yes, sure, that sounds fine, I’d like a 25 quid voucher and then you can’t ever get hold of them because they’re just - then that morning they’ve had a huge fight with their housemates and then they’ve been locked out and oh, just all sorts of crazy things happen where you just—like you almost can’t imagine it (PWYP).

Giving something back to the group in return for their involvement could also include non financial benefits, such as opportunities for training, or the opportunity to have an enjoyable experience. For people who have a more intensive role in the research, for example, young people who join the research team as the public involvement representative, may be motivated to be involved in the research if they can use the experience to develop their curriculum vitae or applications for further education:

But also they’ll want to be able to put stuff for college or university applications, they’ll want to be able to rely on somebody to write them a letter of recommendation, so I think, building that in from the beginning, if you can say, ‘Well, you’re gonna get x amount per session, you’re gonna get to directly feed into x policy and you’ll get something to go into a portfolio for higher education (PWYP).

Ensuring that results of the study are fed back in an accessible way so people understand the value of taking part in research, and that their time was valued could also be important for some:

I know probably a lot of people don’t, people would probably just be like, oh yeah, fine, forget about it, I’ve helped, whatever, but yeah, I like hearing about it. Like with that kind of blood thing, you know, that’s always the most satisfying part of doing that… it’s nice to kind of be like down the line, because you forget about it, don’t you? You do something good and then it’s gone and then, but to actually have something down the line of like, oh, just letting you know actually these were the outcomes, this is what’s come out of this, that’s quite nice (YP).

## Discussion

Through interviews with young people and representatives that work with young people, this research explored barriers to engagement in rapid co-production of public health interventions and suggestions for how we may overcome them. Barriers include young people not being given the opportunity to be involved, with methods of recruitment which are not always designed for, or designed to reach, young people from underserved communities. Research practices and how we communicate about public health research were considered to be intimidating and incompatible with how young people typically communicate. Many young people, particularly those from the most disadvantaged backgrounds cannot prioritise research or public health due to systemic and structural barriers and challenges they face on a daily basis. Mistrust of the government, adults, or any form of authority (including Universities) can further reduce motivation to contribute toward research that could impact public health policy. This is particularly important if research is seen as funded by the government. Adapting research practices and governance so that they are more flexible, inviting, accessible and acceptable to young people is essential for including diverse voices in rapid intervention development projects.

Despite a large literature regarding inclusive co-production [3, 4], there remains a lack of consistency in what it means and how best to do it. This results in confusion among research teams, communities, and populations, and many people still not being given equitable options to take part in research – particularly in situations in which interventions are required very rapidly. Much of the existing advice focuses on establishing long term relationships with communities and community organisations, but this may not be suitable for all populations, particularly those that do not engage with community organisations or do not feel any particular connection to their community. Identification of key individuals, who may not be typical of the population, but can provide access to such participants, is important [20]. In line with previous research [21], we suggest that increasing diversity within research teams is also essential in facilitating identification of and access to diverse communities.

This research has identified barriers to inclusion in research that cannot easily be overcome. For example, some of the most disadvantaged young people face considerable challenges on a daily basis. Many young people did not feel that they would be listened to, and described a mistrust of authority – as well as research and research teams that could potentially influence government policy in a way that may adversely affect them. Trust is further damaged through certain research practices; for example, research teams attempting to rush data collection, or conducting “helicopter research” during which research teams collect data and never contact communities again [22]. Developing and sustaining long-term relationships and investing in populations [23, 24], and those who work with the populations, is essential. In order to build relationships, it is important to acknowledge the barriers faced, build confidence through providing ongoing and sustained training and opportunities to be involved in research [23, 24], and to work with the young people to provide a safe space within which they feel comfortable sharing their views and experiences [25].

This research has also exposed tensions between the increasingly bureaucratised and formal approaches to research governance (which is usually top down and not what communities want), and “best practice” for inclusivity [26]. Use of information sheets, informed consent forms, topic guides that are not shared with participants and recorded conversations contribute to an uncomfortable feeling of formality and the idea that they may be asked questions they cannot answer, and this can result in increased anxiety among individuals. The bureaucratisation of research governance has led to the process becoming more concerned with demonstrating accountability and creating auditable documentation like detailed participant information sheets and consent forms [27]. These formal approaches may be very important when consenting participants to an experimental trial of a new medical treatment, but, as demonstrated here and elsewhere [28], they can contribute to the exclusion of marginalised groups, which is unethical. Other studies with marginalised groups have used less formal approaches to achieving informed consent, including rapport building in advance and informing verbally and discursively, taking consent verbally and treating consent as ongoing and iterative [29, 30]. Recruiting appropriately skilled researchers, particularly those with relevant lived experience, can put participants at ease and facilitate engagement with communities. Allowing participants to select from a range of options in terms of how they share their views in a range of culturally appropriate way can also be useful [31]. There is evidence that conducting interviews with young people using a form of electronic written messaging (texts, WhatsApp, email) is more comfortable for them and has better retention [32, 33]. The current research highlights an urgent need for ethical committees for health research to include consideration of whether formal (auditable) informed consent or other procedures might exclude marginalised populations from participating in research and to support the use of more appropriate methods.

A key strength of this research is that we were able to be adaptable and be responsive to meet the needs of the population. This included tailoring the approaches to recruitment and engagement to the diverse needs of young people. For example, in response to young people highlighting the lack of trust in research teams, we then asked participants if they would be willing to share a modified version of the recruitment advert with those who would not typically respond to, or take part in University/government funded research. At the same time, we adapted our data collection methods to include asynchronous and non verbal approaches to data collection. Being informed about the research by trusted individuals, paired with flexibility in data collection approaches was successful in overcoming some of the frequently mentioned barriers to engagement.

There are a number of limitations to this work. Despite our best efforts, key voices may be missing from the analysis. Most people identified as female, so future work is needed to explore the views of other genders. Furthermore, our approach of asking participant to share study adverts with their peers would have excluded those who are isolated or do not mix with others. Due to time and resource limitations, it was not possible to facilitate inclusion of those who do not speak any English in this project. Future work could attempt to identify acceptable and feasible ways of ensuring those who do not speak English or are socially active can be included in research.

## Conclusions

During public health emergencies, interventions are needed rapidly. Young people from underserved communities are often unable to share their views and experiences because they are not given the opportunity to take part. Furthermore, previous experience of prejudice and discrimination among young people from underserved communities has resulted in a situation in which many people mistrust organisations such as educational establishments and government officials. Complex, exclusive, and inflexible research practices further prohibit recruitment and engagement. Working with communities to co-produce methods of involvement and recruitment, increasing diversity of research teams, simplifying research procedures and offering flexible approaches to data collection may be beneficial.

### Electronic supplementary material

Below is the link to the electronic supplementary material.

Supplementary Material 1

## Data Availability

The datasets used and/or analysed during the current study are not publicly available due to concerns of privacy and confidentiality.
