# Supplementary material for: Engagement in rapid public health research among young people from underserved communities: maximising opportunities and overcoming barriers
Source: BMC Public Health. 2024 Aug 14;24:2217. doi: 10.1186/s12889-024-19762-6 (PMC11325622; doi:10.1186/s12889-024-19762-6)
Supplement: Supplementary file 1 — Supplementary Material 1 [file 12889_2024_19762_MOESM1_ESM.docx]

**Research question**

How can we maximise recruitment and inclusion of diverse participants when doing co-design within very short time frames for emergency responses?

**Introduction**

Thank you for agreeing to take part in this interview. Before we start, I’d just like to remind you that all information that you give will be confidential. Although I would like to record this conversation, only myself and my research team will be able to access the recording. We may publish some of the things you say in academic journals, but it will not be possible to identify you from anything we publish. Do you have any questions before we start?

**Interviews with people from low income households and ethnic minority groups**

***[*** *Interviews with people from low income households and ethnic minority groups will explore views of approaches to recruitment and engagement, with a particular focus on their use to co-develop communication strategies/messages during emergency situations. We will focus on potential problems or barriers and aim to elicit suggestions regarding how we may improve recruitment and engagement in the future. ]*

Brief overview of co-design and the importance of rapid co-design (e.g., during the pandemic/ emergency situations)

**Possible approaches to recruitment**

1. Can you think of any reasons that you or people from your community may not want to help us create/comment on messages?
   1. Are there any groups in particular that you think are more/less likely to want to be involved?
2. What (if anything) do you think we could do to encourage/support people from your community to help us create messages?
3. What do you think would be the best way(s) for us to invite people from your community to help us create /comment on any public health messages?
   1. What would be the best way to tell people about/invite people from your community to help us create/provide feedback on any messages if we had lots of time?
   2. What would be the quickest way?
   3. (if different) what groups/individuals might this faster route miss?

**Perceptions of possible recruitment approaches [if not spontaneously mentioned]**

1. How would you feel about taking part in (co-design) research that you saw advertised on social media?
2. Prompt – Facebook/ Instagram/ tiktok/ Twitter? – others?
3. Are there any platforms /groups/pages that we could / should use to advertise in the future?
4. Are there any platforms /groups/ pages that we should avoid?
5. Can you think of any problems/benefits to this approach? (e.g., concerns about scams etc)
6. Can you think of any ways in which we can overcome any problems?
7. Do you (personally) attend/have contact with any community groups on a regular basis?
8. Which groups?
9. What would be the best way for us to use these groups to invite people to help us create messages?
10. What should we avoid?
11. How would you feel about representatives from these community groups sharing adverts inviting you to help us with our research on our behalf?
12. How would you feel about a researcher attending these groups so that we could let you know about opportunities to work with us?
13. How would you feel about being contacted through UKHSA/ other government officials and invited to help us create messages – by phone? Email? Text/WhatsApp?
14. Can you think of any problems with this approach?

**Engagement and message creation**

1. How do you usually/ most often communicate with friends and family?
   1. What (if any) technology / platforms do you regularly use?
2. There are various ways in which you could help us create messages (e.g., you could join our research team, you could tell us what we need to write, you could help us write the messages, and/or you could give us feedback. How/to what extent would you like to be involved?
   1. Can you think of any other ways in which you could work with us to create messages?
   2. How/to what extent do you think others from your community would like to be involved?
3. If you were to help us create messages/ give feedback on messages how would most like to do so? (e.g., talking to us in person, via phone, text message etc)
4. What do you think would be the best way(s) for us to work with members of your community to create public health messages? (e.g., face to face, technology etc)
5. [if not spontaneously mentioned] How would you feel about giving feedback through the following:
6. Virtual feedback e.g., through WhatsApp/ text message / social media
7. Video / voice message feedback
8. Taking part in an interview with community group leaders

**Recruitment / representation of others**

1. Can you think of anyone within the community who may be willing/able to speak on behalf of people who do not want to take part in research?
2. Can you think of anyone within the community who may be willing/able to collect some data from people who may not want to (directly) take part in research?

**Interviews with community group leaders and representatives**

*[Topic guides will explore how community groups currently communicate and engage with community members, who these communications are likely to reach (or miss), and possible alternative recruitment and engagement approaches]*

Brief overview of co-design and the importance of rapid co-design (e.g., during the pandemic/ emergency situations)

1. Can you tell me about the main audience / scope of community group that you represent? (e.g., age range / location / other characteristics etc)
2. Are there any (sub) groups within your community who you communicate more/ less than others?
   1. Who / which populations do group leaders have more/ less regular contact with?
3. How and how often do you/your community group communicate with group members?
   1. Is technology used to communicate with your group members?
   2. [if yes] What platforms/ types etc?
   3. Which are most helpful/successful in connecting with group members?
   4. What are the benefits and barriers of using this approach to communicate with the group?
4. Can you think of anything that could be done to improve communications/engagement with people in your community?

**Possible approaches to recruitment**

1. Can you think of any reasons that people from your community may not want to help us create/comment on messages?
   1. Are there any groups in particular that you think are more/less likely to want to be involved?
2. What (if anything) do you think we could do to encourage/support people from your community to help us create messages?
3. What do you think would be the best way(s) for us to invite people from your community to help us create /comment on any public health messages?
   1. What would be the best way to tell people about/invite people from your community to help us create/provide feedback on any messages if we had lots of time?
   2. What would be the quickest way?
   3. (if different) what groups/individuals might this faster route miss?
   4. Feasibility of using the group communication channels for research recruitment?
   5. How? Face to face?
   6. What limits/ rules would need to be put in place?

**Engagement and message creation**

1. What do you think would be the best way for us to work with people from your community to create messages? (e.g., talking to us in person, via phone, text message etc)
2. [if not spontaneously mentioned] How feasible would it be to ask people to help us create messages through the following:
3. Virtual feedback e.g., through WhatsApp/ text message / social media
4. Video / voice message feedback
5. Taking part in an interview with community group leaders

**Recruitment / representation of others**

1. Would you or anyone else within the community who may be willing/able to speak on behalf of people who do not want to take part in research?
2. Would you or anyone else within the community who may be willing/able to collect some data from people who may not want to (directly) take part in research?
